# Virtual Exercise in Medicine: A Proof of Concept in a Healthy Population

**DOI:** 10.2196/45637

**Published:** 2024-01-22

**Authors:** Barbara Le Roy, Charles Martin-Krumm, Charlotte Poupon, Raphaëlle Richieri, Eric Malbos, Fanny Barthélémy, Eric Guedj, Marion Trousselard

**Affiliations:** 1 Unité neurophysiologie du stress Institut de recherche biomédicale des armées Brétigny-sur-Orge France; 2 Adaptation, mesure et évaluation en santé. Approches interdisciplinaires Metz France; 3 Vulnérabilité, capabilité, rétablissement Unité religion, culture et société Ecole des psychologues praticiens Paris France; 4 École nationale supérieure de création industrielle Paris France; 5 Département psychiatrie Hôpital de la Conception Institut Fresnel Marseille France; 6 Département de médecine nucléaire Centre européen de recherche en imagerie médicale Institut Fresnel Marseille France; 7 Service de santé des armées Paris France; 8 Réseau ABC des Psychotraumas Montpellier France

**Keywords:** countermeasures, mental health, physical activity, virtual reality, user experience

## Abstract

**Background:**

Science is beginning to establish the benefits of the use of virtual reality (VR) in health care. This therapeutic approach may be an appropriate complementary treatment for some mental illnesses. It could prevent high levels of morbidity and improve the physical health of patients. For many years, the literature has shown the health benefits of physical exercise. Physical exercise in a VR environment may improve the management of mild to moderate mental health conditions. In this context, we developed a virtual environment combined with an ergocycle (the augmented physical training for isolated and confined environments [APTICE] system).

**Objective:**

This study aims to investigate the impact of physical exercise in a VR environment.

**Methods:**

A total of 14 healthy participants (11 men and 3 women; mean age 43.28, SD 10.60 years) undertook 15 minutes of immersive physical exercise using the system. Measures included mindfulness and immersion disposition, subjective perceptions of sensory information, user experience, and VR experience (ie, psychological state, flow, and presence).

**Results:**

First, the APTICE system appears to be a useful tool because the user experience is positive (subscales in the AttrakDiff questionnaire: *pragmatic quality*=0.99; *hedonic quality–stimulation*=1.90; *hedonic quality–identification*=0.67; *attractiveness*=1.58). Second, the system can induce a positive psychological state (negative emotion, *P*=.06) and an experience of flow and presence (*P* values ranging from <.001 to .04). Third, individual immersive and mindful disposition plays a role in the VR experience (*P* values ranging from <.02 to .04). Finally, our findings suggest that there is a link between the subjective perception of sensory information and the VR experience (*P* values ranging from <.02 to .04).

**Conclusions:**

These results indicate that the device is well accepted with positive psychological and exteroceptive outcomes. Overall, the APTICE system could be a proof of concept to explore the benefits of virtual physical exercise in clinical medicine.

## Introduction

The Roman poet Juneval wrote “mens sana in corpore sano” (a healthy mind in a healthy body) [1]. Still relevant today, it has never made as much sense. The body and the mind seem to be indivisible, truly part of a whole [2].

### Virtual Reality

In recent years, virtual reality (VR) has been recognized as a new approach to health [3-6] that seeks to connect the body and mind [7]. The term was first used by Jaron Lamier in 1986 to refer to an advanced technological interface in which the user interacts with a 3D environment that is generated by a computer to simulate real-world experiences [8,9]. The tool can simulate reality and stimulate the body’s senses in ways that are only limited by our imagination. It creates a new space-time that is halfway between the real and the unreal, pushing back the boundaries of reality and experimenting with new paradigms that we would not otherwise have access to [10,11]. Thus, VR goes beyond a simple simulation of the external world. The modulation of interoceptive, exteroceptive, and vestibular information leads the participant to create a representation of their own body. This conceptualization is described as the *body matrix*, which refers to the multisensory representation of the body in the brain and the space directly around the body [12]. Through VR, it is possible to induce the illusion of being and moving in a fake body. This interstice allows individuals to perceive, interpret, and interact with their environment through an internal representation of the world [13]. Repeated VR use may stimulate changes in the brain based on neuroplasticity mechanisms [14]. Riva et al [7] noted that the effects may be heightened by immersive VR systems and the induced sense of presence in the surrounding virtual environment.

*Immersion* is a characteristic of VR systems and is created when the virtual environment replaces the user’s sensory stimuli with virtual sensory stimuli. Through immersion, it is possible to induce the sense of *presence*. Multisensory integration generates a feeling of *being there* and can sometimes lead to the illusion of being in an alternative body [15,16]. Slater [17] defined presence as “the strong illusion of being in a place despite the sure knowledge that you are not there.” Thus, participants have the strong illusion of being in the virtual environment and being able to perceive what is happening in it such as the virtual precipice. However, they consciously know that this is only a perceptual illusion not a reality [18]. Presence is related to *flow*, which refers to “the holistic sensation that people feel when they act with total involvement” [19]. It is a psychological state corresponding to enjoyment, cognitive absorption, and distortions in time perception. The literature on VR highlights the influence of immersion*,* induced by VR systems, on both presence and flow in the virtual environment [20,21].

Nevertheless, interindividual differences have been noted regarding both presence and flow. One relevant factor is mindfulness disposition (MD). MD is characterized by the awareness that emerges when paying purposeful attention to the present moment and responding nonjudgmentally to the unfolding experience [22,23]. It is associated with a protective function in both a healthy population and among patients [24,25]. A recent study by Lefranc et al [26] highlighted that high MD is associated with better positive emotions, interoception, and subjective extrasensory acuity. Top-down conceptual representations and bottom-up multisensory inputs contribute to body awareness. Moseley et al [12] suggested that these representations be integrated with exteroceptive data in the body matrix.

Over the years, VR has become increasingly accessible. It has been particularly beneficial in the field of medicine, whether in the context of medical training, surgery, the treatment of certain neurodegenerative diseases, rehabilitation, pain management, or cognitive and psychological disorders [11,27-34]. The literature shows the value of using VR as a therapeutic tool to treat mental disorders such as anxiety, depression, posttraumatic stress disorder, and phobias [8,31,33-38]. Antidepressants, such as selective serotonin-norepinephrine reuptake inhibitors, or benzodiazepines are the first-line treatment for anxiety symptoms in patients while cognitive behavioral therapy has been found to be effective in reducing them [39-41]. VR interventions such as exposure therapy have been shown to be effective as a coadjutant in mental illness and appear to have the same effects as drug treatments, although the results take longer to become apparent [34]. Used as a complementary therapy, VR may have many advantages, including the ability to recreate a realistic traumatic environment under controlled conditions, which can be complex in vivo [33,42,43]. Most studies show that participants have a high degree of acceptance, and VR use is consistent with postintervention improvements in symptom awareness; a decrease in depressive symptoms; greater motivation to exercise; and better enjoyment, engagement, and affect, particularly in clinical populations [35,37]. VR therapy can stimulate emotion (notably fear), as the participant has the feeling of being present in the unreal environment [18,44]. Thus, it appears to be an innovative nondrug supplement to other treatments that can be demanding for the patient and may have side effects. Although the quality of the technology may play a role in positive outcomes [45], it appears to be an interesting new tool that poses no serious threat to participants [46].

### The Potential of Immersive Physical Activity

In recent years, an increasing body of the literature has investigated the power of immersive physical activity. Physical activity preserves health and protects individuals from many pathologies [47-49]. It can be defined as “any bodily movement produced by skeletal muscles that results in the expenditure of more energy than the resting metabolism” [50]. One of the components of physical activity is physical exercise, understood as “planned, structured, repetitive physical activity whose objective is to improve or maintain one or more components of physical fitness” [50]. For many years, the literature has shown the benefits of physical activity on health, not only physical (ie, reduced mortality, reduced risk of cardiovascular pathologies, reduced incidence of cancer, or weight maintenance) but also cognitive (ie, improved cognitive function, improved sleep, or reduced risk of dementia) and psychological (ie, reduced signs of anxiety and depression or reduced risk of depression), both in the general population (ie, adults, children, and older adults) and in the context of various chronic diseases and health conditions [47-49,51]. However, it is only recently that the scientific community has begun to take an interest in the biological and physiological mechanisms underlying these outcomes [52,53]. People with mental illness often exhibit disrupted sensory processing and perception [54]. Thus, physical activity therapy can be both a physical and psychological countermeasure. However, compliance is a key issue as regular practice is necessary for optimal mental illness management.

Few studies have examined the use of VR in this context, although the pioneering work of Plante et al [55-57] seems to indicate real benefits in terms of well-being, particularly in women [56]. The addition of VR has been found to enhance mood, increase enjoyment and energy, reduce tiredness, enhance motivation and confidence, and increase compliance [57,58]. Enjoyment may play an important role in the benefits gained from exercise [58].

In recent years, there has been an increase in the number of studies that encourage the practice of sports to prevent anxiety disorders and protect against anxiety and depression [59,60]. A recent study demonstrated its importance in the context of the COVID-19 pandemic, where it was able to improve well-being through improved physical and cognitive outcomes and limit psychological disorders related to isolation and confinement [61]. Thus, the literature suggests that VR coupled with physical activity may be a useful way to improve the symptomatology of individuals with anxiety disorders, posttraumatic stress disorder, and depression [61]. Furthermore, many studies have highlighted the ability of natural environments to induce positive emotions, promote well-being, reduce anxiety, improve self-esteem, and reduce negative emotions (ie, fatigue, confusion, tension, depression, and anger-hostility) compared with urban or indoor environments [62,63]. The same observation has been made in VR environments [64]. A virtual environment that offers physical activity in a natural setting seems to have the potential to improve the benefits of VR, especially for people with mental illness [65,66].

### Gaps in the Literature and Objectives of the Study

Many of the systematic reviews and meta-analyses that have been carried out have important limitations, notably related to differences in technology. There is also a lack of longitudinal studies on the long-term effects of VR. Most studies are one-shot experiments that evaluate its benefits before and after the intervention. Evaluation itself is problematic as subjective measures (questionnaires) are typically used and few studies have measured physiological effects (ie, heart rate variability, heart rate, and electrodermal activity). As it can be complex to overcome these gaps, caution is advised in interpreting any results or conclusions [33,35,62,65,67,68]. Given these gaps in the literature, there is a need for more rigorous testing. Any evaluation should be based on three assessment criteria: (1) the activity does not duplicate other countermeasures; (2) it must improve the experience of sport and thus increase its attractiveness (especially relevant for patients with depression) [55,56]; and (3) immersion must provide a multimodal sensory input to the user [69-72]. The benefits of multisensory stimulation have been demonstrated in the context of cognitive and sensorimotor rehabilitation [73] and emotion regulation [10,74].

Thus, the aim of this preliminary proof-of-concept study was to investigate the association between VR and physical exercise in a virtual natural environment to improve the psychological state of healthy participants and the underlying processes, before evaluating its benefits in clinical medicine. We measured the user experience (UX) and evaluated 3 hypotheses:

- Hypothesis 1: positive changes in psychological state are associated with flow and presence during the session in the VR environment.
- Hypothesis 2: both MD and immersion disposition are positively related to change in the participant’s psychological state, flow, and presence.
- Hypothesis 3: there is a relation between the subjective evaluation of sensory information, immersive disposition, and mindful disposition and psychological change.

## Methods

### Ethical Considerations

This study was approved by the Minarm Ethical Committee (N 125 132/MIP/DGA/MINARM). Written consent was obtained from all participants in accordance with the Declaration of Helsinki and subsequent amendments.

### Participants

In total, 14 volunteers (3 women and 11 men), who were declared medically fit, were recruited during the 3 innovation open days at the French Armed Forces Biomedical Research Institute in 2019. They ranged in age from 22 to 59 (mean 43.28, SD 10.60) years and were either working for the French Armed Forces Biomedical Research Institute (n=9) or the French Football Federation (n=5). See Table 1 for the demographic information. The participants were recruited by email and contacted to determine whether they met the inclusion and exclusion criteria. If eligible, they were assigned an appointment for the laboratory session. All participants were asked to abstain from exercise on the day of their participation to ensure that the results were due to the experiment. The inclusion criteria were based on the following: affiliation to a health care system (social security); age between 18 and 75 years; and no history of neurological or cardiovascular disease, diabetes, or medications that could affect the response. Exclusion criteria included pregnancy, the presence of a contraindication to VR (people who had experienced anxiety or nausea during a VR experience, photosensitive epilepsy, vestibular disorder, or severe myopia >3.5 diopters).

**Table 1:** Sociodemographic characteristics of participants (N=14).

Characteristics			Values
Age (y), mean (SD)			43.28 (10.60)
**Gender, n (%)**			
	Men	11 (78.57)	
	Women	3 (21.42)	
**Screen time, mean (SD)**			
	Professional	300.00 (164.73)	
	Personal	111.42 (63.95)	
**Physical activity, n (%)**			
	Yes	10 (71.42)	
	No	4 (28.57)	
**Video games, n (%)**			
	Yes	4 (28.57)	
	No	8 (57.14)	
**Ocular correction, n (%)**			
	Yes	10 (71.42)	
	No	4 (28.57)	

Participants who practiced a physical activity or engaged in video games completed the Addictive Intensity Evaluation Questionnaire (AIEQ). The analysis found that 10 out of 14 (85%) participants engaged in physical activity (mean 31.00, SD 6.20) and 4 (35%) played video games (mean 28.20, SD 14.34). No addictive behaviors were found among the participants in either of these modalities.

### Augmented Physical Training for Isolated and Confined Environments

**Figure 1:**
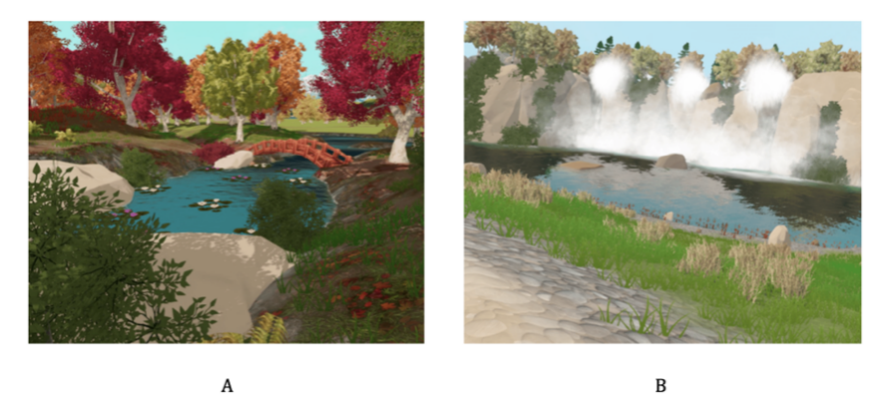
Natural virtual environment images. (A) Forest with stretches of water. (B) Mountain plain with sheep.

This proof-of-concept study is based on the augmented physical training for isolated and confined environments (APTICE) system. The aim of the system is to use physical exercise in a VR environment to improve the well-being of patients with depression. It is composed of a VR‐enabled cycle ergometer (VirZOOM Bike Controller) and a VR-based head-mounted display (Oculus Rift CV1, Oculus VR), which provides visual and auditory inputs. The VR application was developed by GAMIT (Petit-Quevilly) and ran on an Asus A15 TUF566IU-HN326T laptop with an AMD Ryzen 5 4600H 16 GB processor, a 512 GB solid state drive, and an Nvidia GeForce GTX1660 Ti 6 GB graphics card. The VR environment consisted of natural areas of forests and mountain plains (Figure 1). See Multimedia Appendix 1 for further images of the APTICE device.

### Data Collection

#### Population Overview

A 7-item sociobiographical questionnaire was developed to collect standard sociodemographic data such as gender, age, hobbies, physical activity, video game use, and VR experience. The AIEQ evaluated addictive practices [75]. Two versions were used: the 14-item AIEQ-g that measures the intensity of video game playing and the risk of its problematic use and the 14-item AIEQ-s that measures sports practice and the risk of its problematic use.

#### UX of the APTICE Device

The UX of the APTICE device was assessed using the 10-item AttrakDiff questionnaire, which evaluates the hedonic and pragmatic qualities of interactive systems [76]. It is divided into 4 subscales: pragmatic quality, hedonic quality–identity, hedonic quality–stimulation, and attractiveness. Values close to the mean (from 0 to 1) are considered standard values. They indicate that the device meets its objectives with no negative impacts on the user.

#### Psychological Questionnaires

Two questionnaires were used to evaluate psychological dispositions. The 14-item Freiburg Mindfulness Inventory was used to measure MD [77]. It is divided into 2 subscales: acceptation and presence. Immersion disposition was assessed using the 18-item immersive tendencies questionnaire, which is divided into 4 subscales: focus, involvement, emotions, and games [78]. Two questionnaires were used to evaluate psychological state. First, the 12-item Scale of Positive and Negative Experience (SPANE) questionnaire assessed subjective feelings of well-being [79]. The overall scale is divided into 2 subscales: positive and negative emotions. Second, the 20-item Activation-Deactivation Adjective Checklist (AD-ACL) evaluates the level of awareness and emotional disposition [80]. This is divided into 2 dimensions: energic arousal (from energy to tiredness) and tense arousal (from tension to calmness). The energic arousal is further divided into 2 subscales—general activation and deactivation—while the tense arousal is subdivided into general tenseness and calmness.

#### Subjective Evaluation of the Quality of Sensory Information

We developed the Personal Evaluation of Six Senses questionnaire to assess subjective perceptions of vision, sound, touch, olfaction, taste, and equilibrium. Participants evaluated the accuracy of their perceptions from each of their 6 senses using a ranked scale running from 1 to 6.

#### The VR Experience

The VR experience was assessed using the 12-item Educational Flow Questionnaire (EduFlow2), which measures flow [81]. It is divided into 4 dimensions: cognitive control, immersion and time transformation, loss of self-consciousness, and autotelic experience. Cognitive absorption (a summary of the first 3 dimensions) was added as the fourth scale. The 24-item Presence Questionnaire assessed presence [82]. It is divided into 7 subscales: realism, possibility of action, quality of interface, possibility of examination, self-evaluation of performance, sounds, and haptic. APTICE device sickness was assessed using the 16-item Simulator Sickness Questionnaire [83]. It is divided into 2 subscales: nausea and oculomotor.

### Procedure

**Figure 2:**
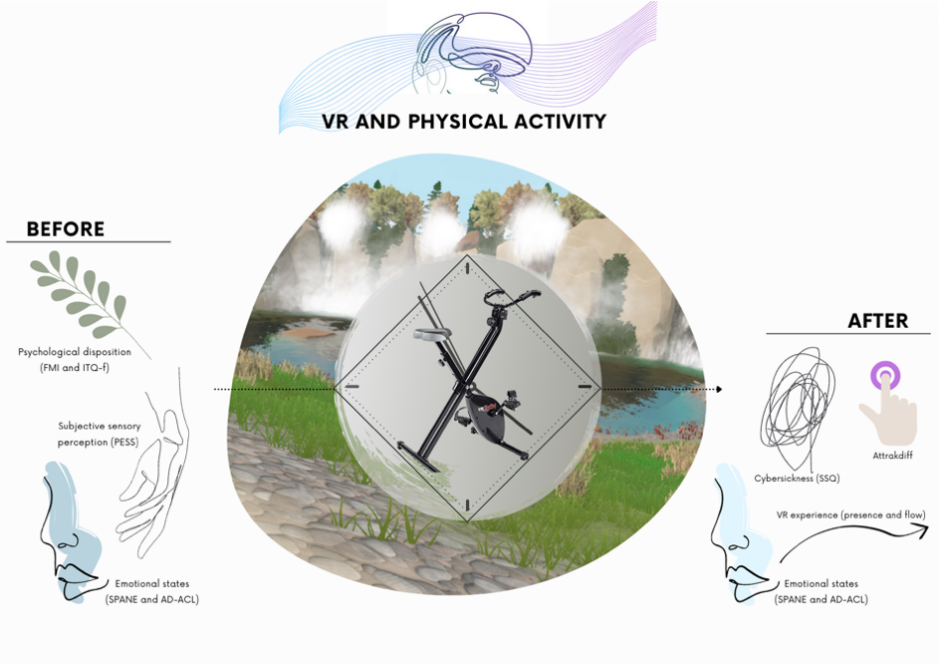
The augmented physical training for isolated and confined environments (APTICE) experimental protocol. AD-ACL: Activation-Deactivation Adjective Checklist; FMI: Freiburg Mindfulness Inventory; ITQ-f: Immersive Tendencies Questionnaire; SPANE: Scale of Positive and Negative Experience; SSQ: Simulator Sickness Questionnaire; VR: Virtual Reality.

The experimental protocol is illustrated in Figure 2. Upon arrival, the participants were asked a few questions to ensure they met the inclusion criteria and signed the consent form. They then completed a series of questionnaires in the following order: sociobiographical questionnaire, AIEQ-g, AIEQ-s, Freiburg Mindfulness Inventory, Personal Evaluation of Six Senses, 18-item immersive tendencies questionnaire, SPANE, and AD-ACL. Then, they engaged in a moderate-intensity bout of exercise in a natural environment for 15 minutes while wearing the VR headset. They could choose their trajectory along various predefined paths and, by turning their head, obtain a 360° view of the virtual environment. At the end of the session, they were asked to complete another series of questionnaires in the following order: SPANE, AD-ACL, EduFlow2, 24-item Presence Questionnaire, AttrakDiff, and Simulator Sickness Questionnaire.

### Statistical Analysis

Statistical analyses were performed using the RStudio software (version 1.2 5001). Descriptive statistics are expressed as mean (SD). The Shapiro-Wilk test was used to determine whether the data were normally distributed. The effects of the APTICE device experience on emotional and activation-deactivation states were assessed as follows: a *t* test (2-tailed) for pre-post comparisons and parametric data or the Mann-Whitney U test for nonparametric data. Kendall correlations were run to explore the relationship among virtual exercise, subjective sensory accuracy, and VR experience. For all analyses, significance was set at *P*<.05. Trends were considered when .05<*P*<.10. Deltas were calculated to compare the temporal impact of the experience measured using the SPANE questionnaire and the AD-ACL.

## Results

### The UX

**Figure 3:**
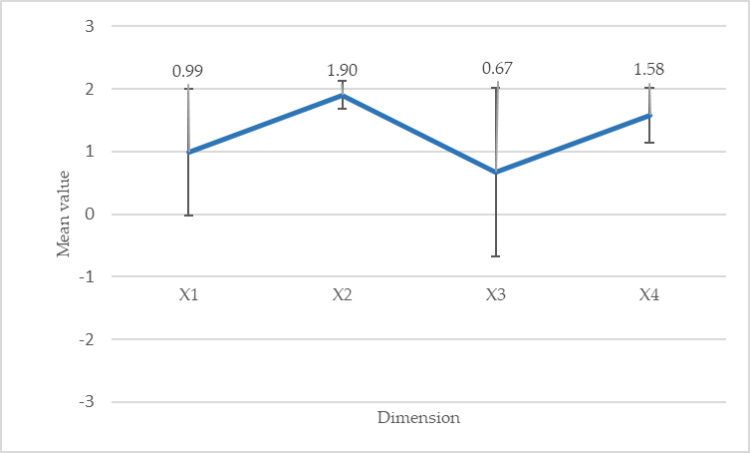
AttrakDiff’s subscales. Values close to the mean (from 0 to 1) are considered standard and indicate that the device meets its objectives with no negative impacts on the user. However, they also suggest that improvements could be made to the system to obtain high positive values. Values outside this neutral zone are considered positive (1-3) or negative (−1 to −3). X1: pragmatic quality; X2: hedonic quality (stimulation); X3: hedonic quality (identification); X4: attractiveness.

The APTICE tool was assessed in terms of UX. Participants reported a positive experience measured as pragmatic quality, hedonic quality–stimulation, hedonic quality–identification, and attractiveness (Figure 3). The scores were particularly high for hedonic quality–stimulation and attractiveness. No participant reported any cybersickness.

### Relationships Between Psychological Assessments, Exteroception, and VR Experience

#### Overview

Multimedia Appendix 2 summarizes the significant correlations between the tested variables.

#### Hypothesis 1: The VR Experience (Change in Psychological State, Flow, and Presence)

The analysis of emotional and arousal states only identified a trend for negative emotions. Participants tended to have fewer negative emotions after the APTICE experiment (t_12_=2.06, *P*=.06).

There were significant positive and negative correlations between flow and presence. Participants who scored high for *possibility to examine* also scored high for *flow cognitive control* (*τ*=0.45, *P*<.001), *flow cognitive absorption* (*τ*=0.67, *P*=.001), and *flow immersion and time transformation* (*τ*=0.55, *P*=.01). Participants who scored high for *possibility to act* also scored high for *flow cognitive absorption* (*τ*=0.58, *P*=.004), *flow cognitive control* (*τ*=0.76, *P*<.001), *flow immersion and time transformation* (*τ*=0.58, *P*=.006), and *flow-autotelic experience* (*τ*=0.58, *P*=.001). As *realism* increased, *flow cognitive control* also increased (*τ*=0.52, *P*=.01). However, as *haptic* increased, *flow loss of self-consciousness* (*τ*=−0.52, *P*=.02) decreased.

Concerning change in psychological states related to flow and presence, our results suggest that there is no correlation between change in emotional state (measured with the SPANE questionnaire) and either flow or presence. However, there were significant negative correlations between flow and changes in activation-deactivation states (measured using the AD-ACL). An increase in *tense activation* (positive delta) was associated with lower scores for *flow immersion and time transformation* (*τ*=−0.46, *P*=.04) and *flow-autotelic experience* (*τ*=−0.52, *P*=.01). No correlation was found between presence and flow, and there were no changes in activation-deactivation.

#### Hypothesis 2: Disposition and the VR Experience (Change in Psychological State, Flow, and Presence)

No relationship was observed between immersive disposition and MD for any subscale.

The analysis found a significant positive correlation between MD and presence. More precisely, higher *MD-acceptation* was associated with a higher score for *possibility to examine* (*τ*=0.49, *P*=.02). There was also a significant positive correlation between MD and flow. High scores for *MD-acceptation* were associated with high scores for *flow cognitive control* (*τ*=0.45, *P*=.03). Finally, there was a significant positive correlation between immersion and flow. Specifically, high scores for *flow loss of self-consciousness* were slightly associated with high scores for *involvement* (*τ*=0.54, *P*=.01).

Concerning disposition and the VR experience, the analysis found no correlation between change in emotional state and either immersive or mindful disposition. Significant positive and negative correlations were found between immersion and change in activation-deactivation. An increase in *tense activation* (positive delta) was associated with higher scores for *games* (*τ*=0.45, *P*=.04). However, an increase in *general activation* (positive delta) was associated with lower scores for *involvement* (*τ*=−0.52, *P*=.02).

#### Hypothesis 3: Subjective Exteroceptive Accuracy, Disposition, and the VR Experience

The analysis found no relation between the subjective exteroceptive evaluation and changes in emotional and activation states, presence, or MD. However, significant positive and negative correlations were observed between immersion and subjective acuity.

**Figure 4:**
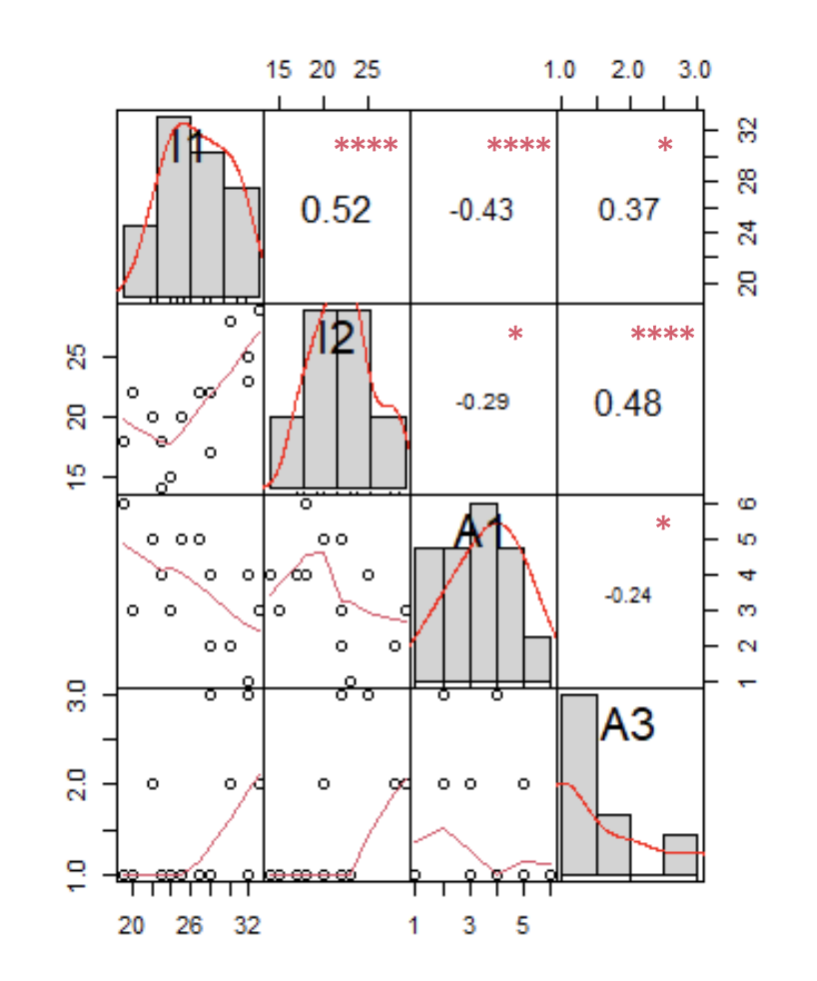
Correlation matrices for immersion and subjective acuity variables. Distributions are shown on the diagonal. Trend curves are shown at the bottom of the diagonal scatter plots. The top diagonal shows correlation coefficients and significance levels. A1: olfaction; A3: vision; I1: focus; I2: involvement. *>.99, **.10, ***.05, ****.01.

Correlation matrices for immersion and subjective acuity variables are shown in Figure 4.

Increased *involvement* was associated with higher subjective *visual acuity* (*τ*=0.48, *P*=.03). In contrast, an increase in *focus* was associated with lower subjective *smell* acuity (*τ*=−0.43, *P*=.04). Low scores for subjective *hearing* were associated with high scores for *flow cognitive absorption* (*τ*=−0.43, *P*=.04) and *flow immersion and time transformation* (*τ*=−047, *P*=.03). Similarly, low scores for subjective *taste* were associated with high scores for *flow cognitive control* (*τ*=−0.49, *P*=.02).

## Discussion

### Principal Findings

The main aim of this proof-of-concept study was to investigate the effect of VR associated with physical activity on the psychological state of healthy participants before further evaluation of patients with depression in a randomized controlled trial. This exploratory study evaluated a new device, named APTICE, which couples physical exercise with a VR headset. This pilot feasibility study proposed variables of interest, which will form the basis for our next randomized controlled trial. The latter will investigate clinical and neurofunctional subtracts in a population affected by depression using VR associated with physical activity. Results from this study provide new insights into the benefits of this type of technology when used in clinical medicine to improve health.

### A Positive UX Experience

As Hassenzahl et al [84] demonstrated, the evaluation of the hedonic and pragmatic qualities of a system is known to influence overall perceptions of its attractiveness. Understanding the UX is crucial in the design of a new device, which is often ignored. The participants in our study were very positive regarding both the *hedonic quality–stimulation* and the *attractiveness* of the device. However, *pragmatic quality* and *hedonic quality–identification* scores were lower. Furthermore, the responses were the most disparate for these 2 dimensions. *Hedonic quality–stimulation* was associated with ideas such as outstanding, impressive, exciting, or interesting. Although the response to the UX appears to be positive, there is room for improvement. The relatively low scores for *hedonic quality–identification* are not surprising, as this aspect relates to the ability of the system to reflect the user’s identity. Similarly, *pragmatic quality* needs to be improved with a focus on usability. Both the appropriateness of the functionality and its accessibility need further attention. However, this short 15-minute experiment allowed us to conclude that the APTICE system meets its development and quality objectives—specifically, to design a device that supports physical exercise in VR. In the longer term, we will need to consider how to improve it, particularly in light of the technological development that has taken place since its creation.

### Psychological Changes Induced by the APTICE Device

Our main hypothesis was that physical exercise in a VR environment could create a positive experience, measured as psychological and sensory feedback from the participants.

Consistent with the literature, our initial results suggest that the APTICE device experience decreases negative emotions [44,85-89]. However, our first hypothesis (that the APTICE device would induce a positive psychological state and an experience of flow and presence) was only partially confirmed. The literature [7,17] notes that presence and flow are usually positively linked, although a negative correlation has been found between haptic presence and loss of self-consciousness in flow experiments. In the absence of a meaningful haptic system, interactions with objects in the VR environment can widen the gap between actual and virtual realities [90]. In our experiment, haptic feedback from the interaction with the ergocycle did not reflect reality, which suggests its key role in inducing flow. For example, there was no body movement when going around the bends and almost no return on effort. The poor quality of the correspondence between the virtual exercise environment and reality could explain the absence of a change in positive emotions.

Our initial results suggest a close relationship between the quality of the technology and the VR experience. This is all the more important as flow (characterized by a deep involvement and absorption in an activity) promotes a state of inner well-being and positive emotions [91,92]. Overall, our results suggest that practicing a physical activity in a VR setting could be used to improve psychological outcomes. According to previous studies [60,61,66], the APTICE device may have potential benefits for patients, especially those with mental illness. The literature also shows that natural scenes support a positive psychological state both in general [65,86] and in the treatment of mental illness [67,93]. This is in line with the reduction in negative emotions in individuals following our study’s APTICE session. Although APTICE needs improvement, both the positive response to the UX and its effect on the user’s psychological state suggest that regular use may have a positive impact on mental health.

### Relationships Between Disposition and VR Experience

Our results partially confirm our second hypothesis, which focused on the impact of immersive disposition and MD on the VR experience. We found no relationship between immersive disposition and MD in our sample. Immersive disposition is used to evaluate the potential to immerse a subject in a situation, whereas MD is characterized by the ability to be in the here and now. Therefore, it is possible that these 2 dimensions are unrelated. Our experiment showed that the involvement subscale of immersive disposition was associated with a loss of self-consciousness in terms of flow effect. An individual’s interest in a target object [94] or their motivational state in relation to a target object [95] has been described as a condition for flow experience in VR [96]. Furthermore, our experiment found that immersion was unrelated to presence, which conflicts with the literature [15,37]. A key difference compared with earlier work is that our participants were asked to make a physical effort. It is possible that this effort counteracted their immersive disposition. If we turn to the relationship among MD, presence, and flow, acceptance seems to be the most relevant dimension. Acceptance consists of accepting inner events such as emotions, thoughts, or beliefs as they are felt [97]. It does not mean resignation but rather perceiving one’s own experience with an attitude that acknowledges it, rather than judging it as either good or bad. Thus, the ability to accept what is happening now may be a more useful way to examine presence and cognitive control than simply being in the here and now. Collectively, these results suggest that physical exercise in VR may be improved by acceptance, which enhances the feeling of presence.

### APTICE Device and Exteroceptive Modulations

Our final hypothesis concerning the relationship between subjective exteroceptive perceptions of sensory information and physical exercise in the VR experience was exploratory. On the one hand, our results show that there is an assumption that information provided by all 5 senses may help the user to become immersed in the experience of where they are, whom they are with, and what they are doing. The feeling of a real experience gives rise to presence. On the other hand, mindful participants pay more attention to information from their bodies, leading to better adaptation to the environment [98]. Using functional magnetic resonance imaging, Farb et al [99] identified several brain regions associated with mindfulness. In particular, they found that deactivation of the medial prefrontal cortex and increased activation of parietal areas were associated with proprioception and sensory–motor body experiences. Mehling [100] reported the use of external stimulation when attempting to understand how felt sensations are used internally to regulate stress or attention. Such information is integrated and linked to the person’s emotional state as a function of whether the body is experienced as safe [98,100].

Our results suggest that subjective preferences in exteroception-perception are linked to the experience of physical exercise in the VR environment. Furthermore, they show that immersion is correlated with subjective visual acuity. The participants in our experiment were cycled in a virtual environment based on natural visual information. Unsurprisingly, high scores for subjective visual acuity were associated with flow. Many studies have highlighted the potential of external sensory information to enrich the lived experience [101-104]. Exteroception information can generate intense emotional processes [105] and flavor manipulation within VR [101-104]. However, the evidence is weak, and it is also possible that such an environment may inhibit VR experiences because of its limited capacity to provide wider sensory inputs [105]. Another outcome of our study was that individual preferences may play a role in the VR experience. Our findings showed that this experience is negatively associated with all forms of external sensory stimulation (ie, hearing, taste, and smell) except vision. This suggests that other senses are partially inhibited, and only vision is recruited on a large scale. Vision is an essential component of the APTICE experience.

In this context, Slater and Usoh [106] suggested that an individual’s experience is encoded by visual, auditory, and kinesthetic systems of representation. Depending on the context, the person will naturally tend to favor one system over another. However, the latter authors noted that the visual system predominates in individuals who report a higher sense of presence and those who process information in the first person. Thus, individual characteristics may be a key factor in any experiment. Overall, our study suggests that the APTICE system may alter multisensory representations during physical exercise. Future studies should address this issue, which remains unexplored.

### Future Clinical Applications

VR technologies appear to complement established approaches to mental health care. Its association with physical activity makes it an interesting new approach that merits further investigation. Furthermore, the use of VR in health care is expanding rapidly. There are many new opportunities in clinical medicine, including mental illness, where VR may be an alternative treatment [4,5,107,108]. Our findings validated the impact of physical exercise in a VR environment on negative emotions in a healthy population. Although our results should be interpreted with caution, because of the small sample size, they highlight the importance of better understanding the processes involved in healthy participants. Beyond the efficacy of interventions to determine which populations might benefit from VR combined with physical activity, it is important to understand the processes that predispose this state in healthy individuals. Further studies with larger sample sizes are required to evaluate the role of these processes in clinical research. Thus, the next step is to study clinical and neurofunctional subtracts in a population with depression before proposing the tool as a countermeasure (ID-RCB: 2020-A03415-34) for this population and other people in health care. There is an untapped opportunity to use VR as a prevention tool and to target the processes that make an individual poorly adapted to the environment. This is particularly the case for people who work in challenging confined and isolated environments or extreme and unusual environments [65].

### Limitations

This study has 4 main limitations. The first and most important factor is the small sample size. This study was intended to be a pilot feasibility study that will support a future controlled randomized trial. In this context, it validated the usefulness of the APTICE system and highlighted the interaction between the variables of interest. In the next phase of our work, we will launch a larger clinical study of participants with depression. The second limitation relates to the use of subjective self-report measures. An objective sensory evaluation needs to be developed for healthy participants, which would help researchers to better investigate the human-body relationship. Subjective variables should be combined with physiological measures, such as heart rate variability. Third, our results cannot be generalized because the study population was recruited from among armed forces personnel and footballers, who are usually different from the general population in terms of fitness and psychological state. Finally, the last limitation concerns the VR equipment used in our experiment, which is becoming dated. A new version of the Oculus headset is already available, with a better graphics interface.

### Conclusions

This exploratory proof-of-concept study investigated some of the processes implicated in physical exercise in a VR environment with the aim of better understanding their relationship with psychological state in a sample of healthy individuals. It represents the first step in a larger randomized controlled trial that will investigate clinical and neurofunctional subtracts in a population with depression. Our results suggest that the APTICE environment can change negative emotional states, consistent with the experiences of flow and presence. Moreover, our findings demonstrate that immersive and mindful disposition play an important role in the VR experience. Finally, they also suggest that the subjective exteroceptive perception of sensory information may be a key aspect and seems to indicate that one sense may prevail over another at the level of the individual. Our study has several implications for clinical medicine: (1) VR can help enhance and reinforce the beneficial actions of physical activity; (2) APTICE is a promising system and may be effective in improving mental health; and (3) APTICE has the potential to be used as an alternative treatment to drugs and to improve quality of life. However, many questions remain unanswered, and further work is needed to exploit the potential of VR associated with physical activity both as prevention and treatment.

## Appendix

Multimedia Appendix 1 Augmented Physical Training for Isolated and Confined Environments (APTICE) system description.

Multimedia Appendix 2 Significant correlations between tested variables as a function of the 3 hypotheses.

## Abbreviations

AD-ACL: Activation-Deactivation Adjective Checklist
AIEQ: Addictive Intensity Evaluation Questionnaire
APTICE: augmented physical training for isolated and confined environments
MD: mindfulness disposition
SPANE: Scale of Positive and Negative Experience
UX: user experience
VR: virtual reality

## Data Availability

The data sets generated and analyzed during this study are not publicly available because they are the property of the French Armed Forces Health Service. Although data are not available to the public, they are available from author MT upon reasonable request.
