# Supplementary material for: Virtual Exercise in Medicine: A Proof of Concept in a Healthy Population
Source: JMIR Form Res. 2024 Jan 22;8:e45637. doi: 10.2196/45637 (PMC10845022; doi:10.2196/45637)
Supplement: Multimedia Appendix 1 [file formative_v8i1e45637_app1.docx]

**Multimedia Appendix**

APTICE (Augmented Physical Training for Isolated and Confined Environments) system propose a ride in a virtual environment through an immersive device coupled with an ergocycle. Each programmed session lasts approximately 30 minutes. This environment has several possible paths and events that allow to give life to this virtual universe and renew the interest over the sessions. Each user has a dedicated profile that records every data related to the sessions.

APTICE system features include:

- several environments to choose

- a human body in 3D model placed on the bike

- haptic feedback (i.e., vibrations) according to the terrain

- sound feedback of the environment (e.g., waterfall), and sonification of the activity (e.g., increase of the bike mechanism as the speed increases)

- objectives to be completed to unlock new environments

- user profile space (i.e., kilometers ridden, duration, average speed, lap record, top speed, performance of the last five sessions)


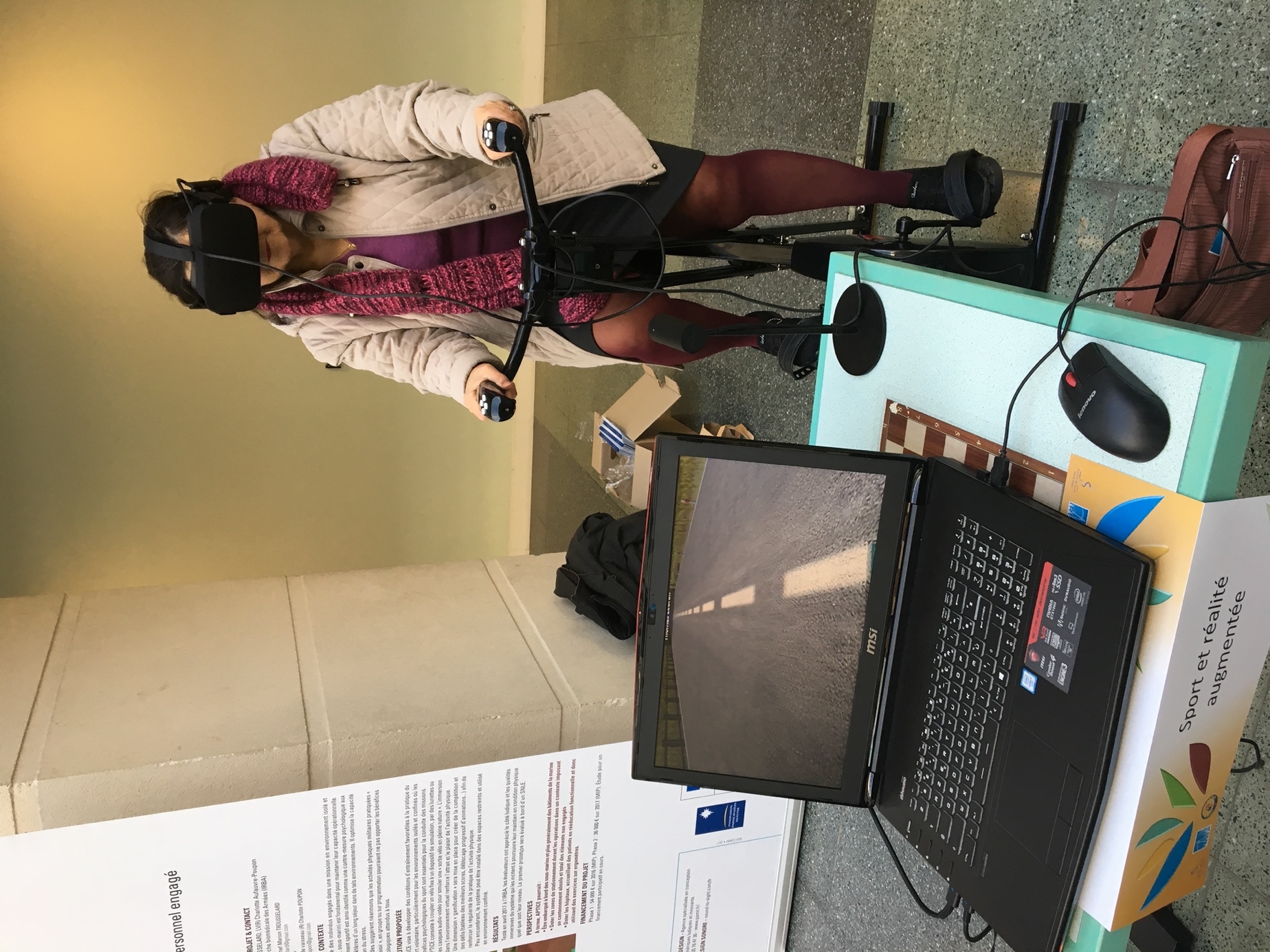


Figure 1. APTICE system test session


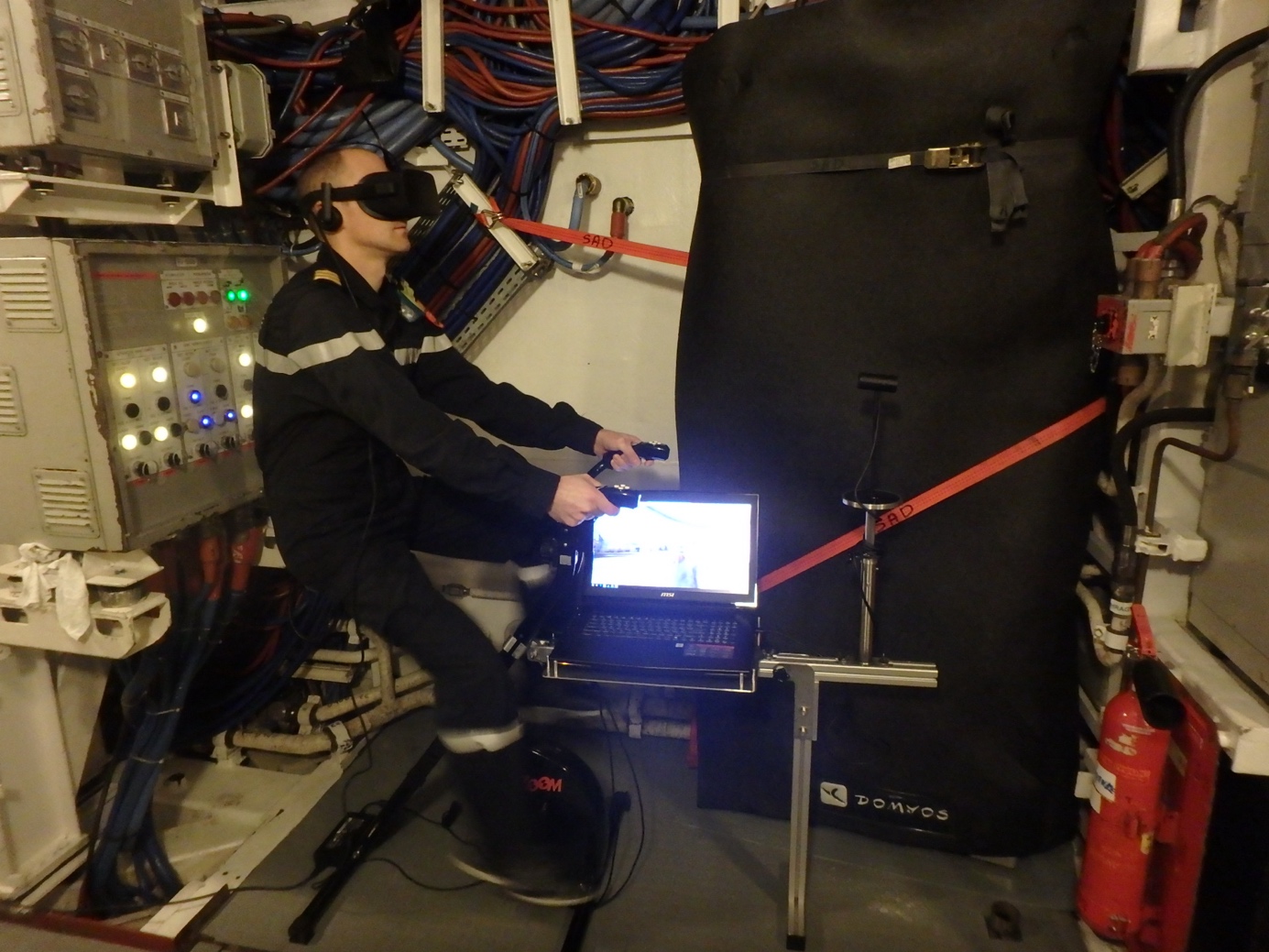


Figure 2. APTICE system on board a submarine


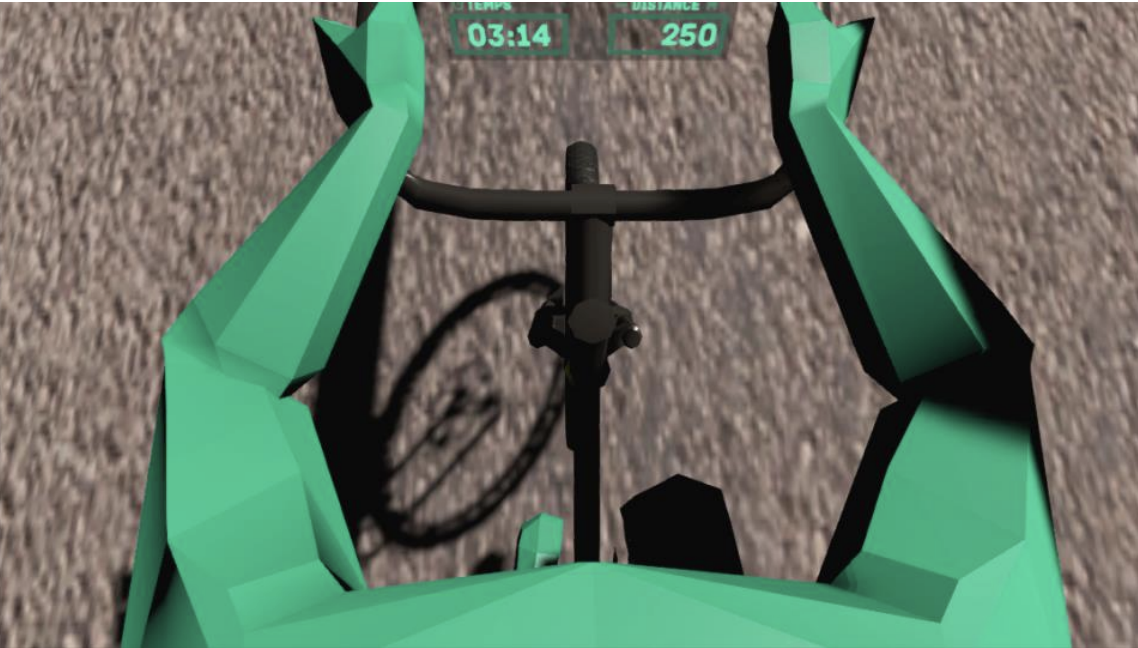


Figure 3. Body awareness representation
