# Supplementary material for: Virtual Exercise in Medicine: A Proof of Concept in a Healthy Population
Source: JMIR Form Res. 2024 Jan 22;8:e45637. doi: 10.2196/45637 (PMC10845022; doi:10.2196/45637)
Supplement: Multimedia Appendix 2 [file formative_v8i1e45637_app2.docx]

**Table S1.** Significant correlations between tested variables as a function of the 3 hypotheses^a,b^.

| Variable | | | Delta AD-ACL tense activation | Delta AD-ACL general activation | FMI acceptation | PQ-f haptic | PQ-f realism | PQ-f possibility to examine | PQ-f possibility to act | Flow-autotelic experience | Flow immersion and time transformation | Flow loss of self-consciousness | Flow cognitive control | Flow cognitive absorption | ITQ-f involvement | ITQ-f games | ITQ-f focus | Vision | Smell | Hearing | Taste |
| --- | --- | --- | --- | --- | --- | --- | --- | --- | --- | --- | --- | --- | --- | --- | --- | --- | --- | --- | --- | --- | --- |
| **Hypothesis 1: the VR experience (change in psychological state, flow, and presence)** | | | | | | | | | | | | | | | | | | | | | |
|  | **Delta AD-ACL tense activation** | | | | | | | | | | | | | | | | | | | | |
|  |  | τ | 1^c^ | —^d^ | — | — | — | — | — | −0.52 | −0.46 | — | — | — | — | 0.45 | — | — | — | — | — |
|  |  | *P* value^b^ | — | — | — | — | — | — | — | .01 | .04 | — | — | — | — | .04 | — | — | — | — | — |
|  | **Delta AD-ACL general activation** | | | | | | | | | | | | | | | | | | | | |
|  |  | τ | — | 1 | — | — | — | — | — | — | — | — | — | — | −0.52 | — | — | — | — | — | — |
|  |  | *P* value^b^ | — | — | — | — | — | — | — | — | — | — | — | — | .02 | — | — | — | — | — | — |
|  | **FMI acceptation** | | | | | | | | | | | | | | | | | | | | |
|  |  | τ | — | — | 1 | — | — | 0.49 | — | — | — | — | 0.45 | — | — | — | — | — | — | — | — |
|  |  | *P* value^b^ | — | — | — | — | — | .02 | — | — | — | — | .03 | — | — | — | — | — | — | — | — |
|  | **PQ-f haptic** | | | | | | | | | | | | | | | | | | | | |
|  |  | τ | — | — | — | 1 | — | — | — | — | — | −0.52 | — | — | — | — | — | — | — | — | — |
|  |  | *P* value^b^ | — | — | — | — | — | — | — | — | — | .02 | — | — | — | — | — | — | — | — | — |
|  | **PQ-f realism** | | | | | | | | | | | | | | | | | | | | |
|  |  | τ | — | — | — | — | 1 | — | — | — | — | — | 0.52 | — | — | — | — | — | — | — | — |
|  |  | *P* value^b^ | — | — | — | — | — | — | — | — | — | — | .01 | — | — | — | — | — | — | — | — |
|  | **PQ-f possibility to examine** | | | | | | | | | | | | | | | | | | | | |
|  |  | τ | — | — | — | — | — | 1 | — | — | 0.55 | — | 0.45 | 0.67 | — | — | — | — | — | — | — |
|  |  | *P* value^b^ | — | — | — | — | — | — | — | — | .01 | — | .000 | .001 | — | — | — | — | — | — | — |
|  | **PQ-f possibility to act** | | | | | | | | | | | | | | | | | | | | |
|  |  | τ | — | — | — | — | — | — | 1 | 0.58 | 0.58 | — | 0.76 | 0.58 | — | — | — | — | — | — | — |
|  |  | *P* value^b^ | — | — | — | — | — | — | — | .001 | .006 | — | .000 | .004 | — | — | — | — | — | — | — |
| **Hypothesis 2: disposition and the VR experience (change in psychological state, flow, and presence)** | | | | | | | | | | | | | | | | | | | | | |
|  | **Flow-autotelic experience** | | | | | | | | | | | | | |  |  |  |  |  |  |  |
|  |  | τ | — | — | — | — | — | — | — | 1 | — | — | — | — | — | — | — | — | — | — | — |
|  |  | *P* value^b^ | — | — | — | — | — | — | — | — | — | — | — | — | — | — | — | — | — | — | — |
|  | **Flow immersion and time transformation** | | | | | | | | | | | | | | | | | | | | |
|  |  | τ | — | — | — | — | — | — | — | — | 1 | — | — | — | — | — | — | — | — | — | — |
|  |  | *P* value^b^ | — | — | — | — | — | — | — | — | — | — | — | — | — | — | — | — | — | — | — |
|  | **Flow loss of self-consciousness** | | | | | | | | | | | | | | | | | | | | |
|  |  | τ | — | — | — | — | — | — | — | — | — | 1 | — | — | — | — | — | — | — | — | — |
|  |  | *P* value^b^ | — | — | — | — | — | — | — | — | — | — | — | — | — | — | — | — | — | — | — |
|  | **Flow cognitive control** | | | | | | | | | | | | | | | | | | | | |
|  |  | τ | — | — | — | — | — | — | — | — | — | — | 1 | — | — | — | — | — | — | — | — |
|  |  | *P* value^b^ | — | — | — | — | — | — | — | — | — | — | — | — | — | — | — | — | — | — | — |
|  | **Flow cognitive absorption** | | | | | | | | | | | | | | | | | | | | |
|  |  | τ | — | — | — | — | — | — | — | — | — | — | — | 1 | — | — | — | — | — | — | — |
|  |  | *P* value^b^ | — | — | — | — | — | — | — | — | — | — | — | — | — | — | — | — | — | — | — |
|  | **ITQ-f involvement** | | | | | | | | | | | | | | | | | | | | |
|  |  | τ | — | — | — | — | — | — | — | — | — | 0.54 | — | — | 1 | — | — | — | — | — | — |
|  |  | *P* value^b^ | — | — | — | — | — | — | — | — | — | .01 | — | — | — | — | — | — | — | — | — |
|  | **ITQ-f games** | | | | | | | | | | | | | | | | | | | | |
|  |  | τ | — | — | — | — | — | — | — | — | — | — | — | — | — | 1 | — | — | — | — | — |
|  |  | *P* value^b^ | — | — | — | — | — | — | — | — | — | — | — | — | — | — | — | — | — | — | — |
|  | **ITQ-f focus** | | | | | | | | | | | | | | | | | | | | |
|  |  | τ | — | — | — | — | — | — | — | — | — | — | — | — | — | — | 1 | — | −0.43 | — | — |
|  |  | *P* value^b^ | — | — | — | — | — | — | — | — | — | — | — | — | — | — | — | — | .04 | — | — |
| **Hypothesis 3: subjective exteroceptive accuracy, disposition, and the VR experience** | | | | | | | | | | | | | | | | | | | | | |
|  | **Vision** | | | | | | | | | | | | | | | | | | | | |
|  |  | τ | — | — | — | — | — | — | — | — | — | — | — | — | 0.48 | — | — | 1 | — | — | — |
|  |  | *P* value^b^ | — | — | — | — | — | — | — | — | — | — | — | — | .03 | — | — |  | — | — | — |
|  | **Smell** | | | | | | | | | | | | | | | | | | | | |
|  |  | τ | — | — | — | — | — | — | — | — | — | — | — | — | — | — | — | — | 1 | — | — |
|  |  | *P* value^b^ | — | — | — | — | — | — | — | — | — | — | — | — | — | — | — | — | — | — | — |
|  | **Hearing** | | | | | | | | | | | | | | | | | | | | |
|  |  | τ | — | — | — | — | — | — | — | — | −0.47 | — | — | −0.43 | — | — | — | — | — | 1 | — |
|  |  | *P* value^b^ | — | — | — | — | — | — | — | — | .03 | — | — | .04 | — | — | — | — | — | — | — |
|  | **Taste** | | | | | | | | | | | | | | | | | | | | |
|  |  | τ | — | — | — | — | — | — | — | — | — | — | −0.49 | — | — | — | — | — | — | — | 1 |
|  |  | *P* value^b^ | — | — | — | — | — | — | — | — | — | — | .02 | — | — | — | — | — | — | — | — |

^a^τ: correlation coefficient.

^b^*P* value significance for correlation analyses.

^c^Not applicable.

^d^Not available.
